# *Vibrio parahaemolyticus* Type VI Secretion System 1 Is Activated in Marine Conditions to Target Bacteria, and Is Differentially Regulated from System 2

**DOI:** 10.1371/journal.pone.0061086

**Published:** 2013-04-16

**Authors:** Dor Salomon, Herman Gonzalez, Barrett L. Updegraff, Kim Orth

**Affiliations:** Department of Molecular Biology, University of Texas Southwestern, Medical Center, Dallas, Texas, United States of America; East Carolina University School of Medicine, United States of America

## Abstract

*Vibrio parahaemolyticus* is a marine bacterium that thrives in warm climates. It is a leading cause of gastroenteritis resulting from consumption of contaminated uncooked shellfish. This bacterium harbors two putative type VI secretion systems (T6SS). T6SSs are widespread protein secretion systems found in many Gram-negative bacteria, and are often tightly regulated. For many T6SSs studied to date, the conditions and cues, as well as the regulatory mechanisms that control T6SS activity are unknown. In this study, we characterized the environmental conditions and cues that activate both *V. parahaemolyticus* T6SSs, and identified regulatory mechanisms that control T6SS gene expression and activity. We monitored the expression and secretion of the signature T6SS secreted proteins Hcp1 and Hcp2, and found that both T6SSs are differentially regulated by quorum sensing and surface sensing. We also showed that T6SS1 and T6SS2 require different temperature and salinity conditions to be active. Interestingly, T6SS1, which is found predominantly in clinical isolates, was most active under warm marine-like conditions. Moreover, we found that T6SS1 has anti-bacterial activity under these conditions. In addition, we identified two transcription regulators in the T6SS1 gene cluster that regulate Hcp1 expression, but are not required for immunity against self-intoxication. Further examination of environmental isolates revealed a correlation between the presence of T6SS1 and virulence of *V. parahaemolyticus* against other bacteria, and we also showed that different *V. parahaemolyticus* isolates can outcompete each other. We propose that T6SS1 and T6SS2 play different roles in the *V. parahaemolyticus* lifestyles, and suggest a role for T6SS1 in enhancing environmental fitness of *V. parahaemolyticus* in marine environments when competing for a niche in the presence of other bacterial populations.

## Introduction

*Vibrio parahaemolyticus* is a Gram-negative halophylic bacterium commonly found in marine and estuarine environments under warm climate conditions [1]. It is a leading cause of gastroenteritis resulting from consumption of contaminated uncooked shellfish and can also cause wound infections and septicemia [2], [3]. This bacterium is becoming increasingly important as pandemic strains are emerging throughout the world [2], [3], [4]. A better understanding of the *V. parahaemolyticus* virulence mechanisms, as well as mechanisms that enhance its environmental fitness, is essential for better diagnosis, treatment, and prevention of infections.

Several virulence determinants are known for *V. parahaemolyticus*, among them secreted toxins [5] and two type III secretion systems (T3SS) [6]. The genome sequence of *V. parahaemolyticus* RIMD 2210633 also revealed the presence of two putative type VI secretion systems (T6SS) [7] (Figure S1). T6SS1 is found on chromosome 1 (VP1386–VP1414), while T6SS2 is found on chromosome 2 (VPA1025–VPA1046) (Fig. S1). Notably, while T6SS2 is found in all tested strains of *V. parahaemolyticus*, T6SS1 is found predominantly in clinical isolates [8]. Both *V. parahaemolyticus* T6SSs were recently proposed to contribute to adhesion to cultured cell monolayers [8]. However, the functionality of T6SS1 was not demonstrated.

The T6SS is a newly described mechanism for protein transport into a recipient cell, and is implicated in the virulence of various bacterial strains [9], [10], [11]. More recently, several T6SS were shown to target bacterial cells, connecting T6SS with interbacterial competition [12], [13], [14]. The T6SS is composed of 13 ‘core genes’ and additional ‘accessory genes’ [15]. The current model of T6SS secretion is of an exterior sheath, composed of heterodimers of VipA and VipB, that contracts to provide energy needed to translocate an interior tube that is made of Hcp hexamers, and is capped with a VgrG trimer [16], [17].

In many cases, more than one T6SS gene cluster is found in a single bacterial genome [11], and those often possess different functions and regulation mechanisms [14], [18], [19]. While numerous T6SSs in various bacteria have been identified, the environmental conditions and cues activating them, as well as the regulatory mechanisms that control their expression, are described only for a few systems. For example, the *Vibrio cholerae* T6SS requires endocytosis by host cells to secrete VgrG [20]. A *Burkolderia pseudomallei* T6SS was shown to be induced upon invasion of macrophages [21]. The *Agrobacterium tumefaciens* and *Pectobacterium atrosepticum* T6SSs were found to be induced by acidic conditions and potato tuber extracts, respectively, which simulate the conditions found in their plant hosts [22], [23], [24]. Interestingly, quorum sensing is a common regulator of T6SSs in various bacteria [19], as seen with *Vibrio cholerae* [25], [26], *Burkholderia cenocepacia* [27], and *Pseudomonas aeruginosa*, in which the three T6SSs were found to be differentially regulated by quorum sensing [28], [29]. Notably, expression of components from both *V. parahaemolyticus* T6SSs was found to be regulated by the quorum sensing master regulator, OpaR [30], [31]. OpaR, a homolog of the *Vibrio harveyi* LuxR [32], is a transcriptional regulator that is not expressed at low cell densities, and is induced at high cell densities when the concentration of autoinducer molecules is high [33]. The OpaR regulon was reported to also include T3SS1, induction of the capsule, and swarming [30].

To date, only few T6SS secreted effectors have been described and characterized [12], [34], [35], [36], [37], [38]. Among them are VgrG proteins that have an additional effector domain with an enzymatic activity (called ‘evolved VgrGs’) such as actin cross-linking [34] and ADP ribosylation [39]. An additional type VI effector, EvpP, was identified in *Edwardsiella tarda*, but its function and activity are unclear [36]. VasX was found to be a type VI secreted effector of *V. cholerae*, with unknown function that is required for virulence against the eukaryotic host model *Dictyostelium discoideum* [35]. In addition, a large group of bacteriolytic type VI effectors with peptidoglycan amidase activity, and their matching immunity genes, were recently identified in many bacterial species, but were not found in *Vibrios* [37], [38], [40].

In this study, we aimed to characterize the regulatory mechanism and environmental cues that control the *V. parahaemolyticus* T6SSs and to identify their functions. We found that T6SS1 is most active under warm marine-like conditions, while T6SS2 is active under low salt conditions. Surface sensing and quorum sensing differentially regulated both systems. Moreover, we identified an anti-bacterial activity for T6SS1 and showed a correlation between the presence of T6SS1 in environmental isolates and virulence against other bacteria. The activation of the anti-bacterial T6SS1 under warm marine conditions suggests a role for this system in enhancing fitness of clinical isolates in marine environments.

## Results

### *V. parahaemolyticus* T6SS1 is Active Under Marine-like Conditions, while T6SS2 is Active Under Low Salt Conditions

*V. parahaemolyticus* can live in different environments, from marine and estuarine ecosystems, to the animal gut [2], [3], [41]. Therefore, we set out to test various environmental conditions including temperature, salinity, and culture cell densities, to identify the optimal conditions for activation of T6SS1 and T6SS2. To this aim, we monitored the expression and secretion of the T6SS secreted proteins Hcp1 (for T6SS1) and Hcp2 (for T6SS2) containing a C-terminal myc tag fused in-frame with the genomic copies of the *hcp* genes in the *V. parahaemolyticus* RIMD 2210633 derivative strain, POR1 [6]. These strains were grown at variable temperatures (23°C, 30°C, 37°C) in low salt (LB media) or high salt (MLB media) and starting with cultures at high (OD_600_ = 0.9) or low (OD_600_ = 0.18) cell densities.

**Figure 1:**
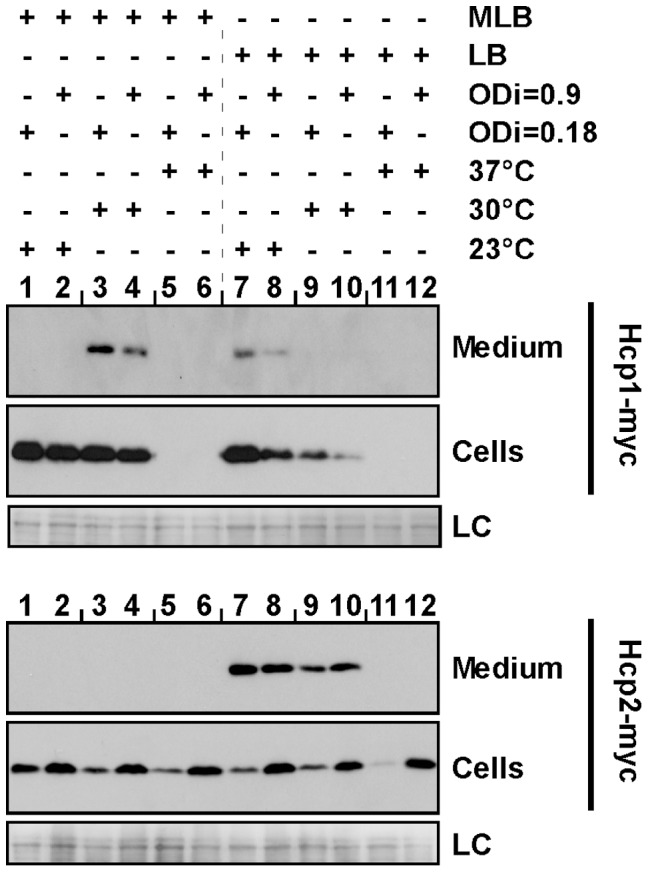
Hcp1 is secreted under marine-like conditions and Hcp2 is secreted under low salt conditions. *V. parahaemolyticus* POR1 strains containing endogenously C-terminal myc-tagged Hcp1 or Hcp2 were grown under the indicated temperature and media conditions at two different initial OD_600_ (ODi). Expression (cells) and secretion (medium) of Hcp1-myc and Hcp2-myc were detected by immunoblot using anti-myc antibodies. Loading control (LC) is shown for total protein lysate.

Hcp1 was expressed at 23°C and 30°C, but not at 37°C, in both high salt (MLB media) and low salt (LB media) conditions, although expression levels were higher in the former (Figure 1, lanes 1–4, 7–10, respectively). However, Hcp1 was only secreted in high salt media at 30°C (Figure 1, top panel lanes 3,4) and to a lesser extent in low salt media at 23°C (Figure 1, lanes 7,8). Remarkably, Hcp1 secretion increased as the salt concentration in the media was higher (Figure S2). Notably, expression and secretion of Hcp1 were higher when the initial cell density of the culture was lower (Figure 1, lanes 3,4,7,8). These results suggest that T6SS1 is most active in high salinity and warm temperature conditions similar to summer marine conditions, and at low cell densities.

Contrary to Hcp1, Hcp2 was expressed at all tested temperatures (23°C, 30°C, and 37°C), while increased expression levels were detected when the initial cell density of the culture was higher (Figure 1). However, Hcp2 was only secreted in low salt media at 23°C and 30°C, but not at 37°C, and secretion levels of Hcp2 were slightly higher at 23°C compared to 30°C (Figure 1, lanes 7–10). These results suggest that T6SS2 is most active at cold and warm temperatures in low salt conditions.

Unless stated otherwise, subsequent assays were performed in MLB at 30°C and low initial cell densities for the T6SS1 Hcp1, and in LB at 23°C and at high initial cell densities for the T6SS2 Hcp2, as these conditions yielded the highest expression and secretion levels for Hcp1 and Hcp2, respectively (Figure 1).

### Quorum Sensing and Surface Sensing Reciprocally Regulate T6SS1 and T6SS2

**Figure 2:**
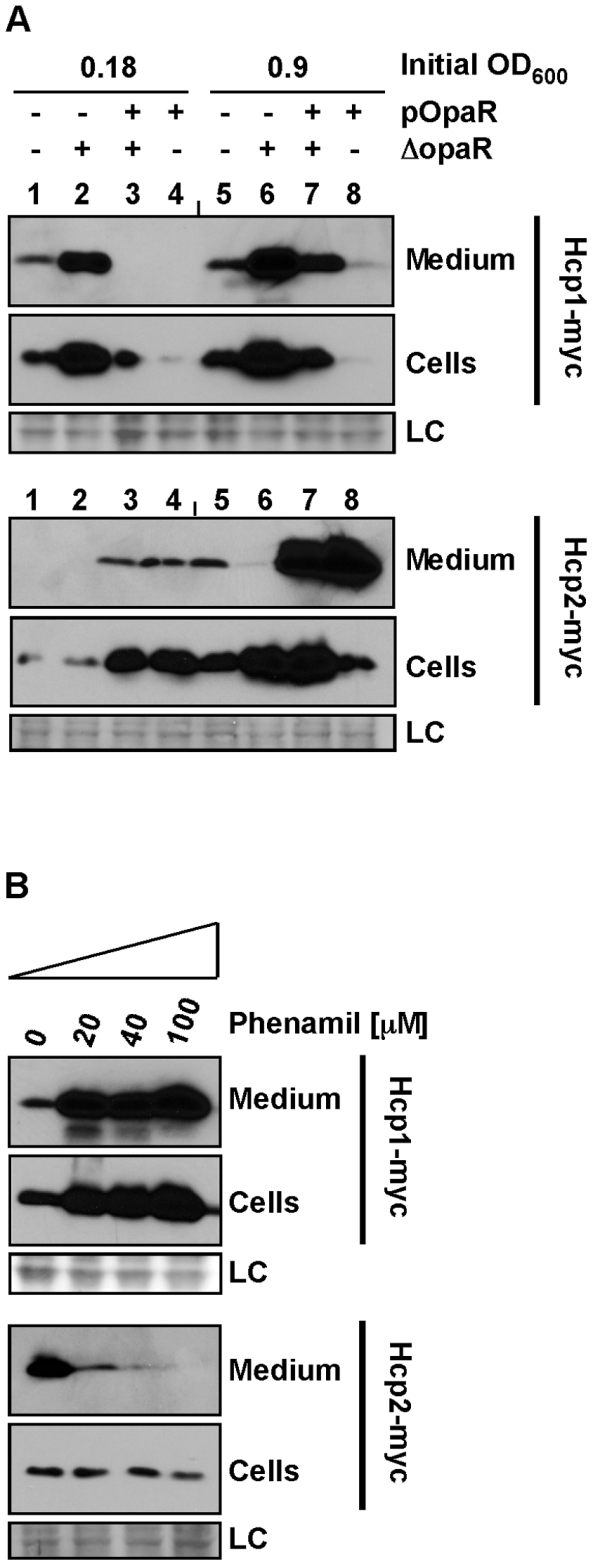
T6SS1 and T6SS2 are regulated by surface- and quorum-sensing. *V. parahaemolyticus* POR1 strains containing endogenously C-terminal myc-tagged Hcp1 or Hcp2 were grown in MLB media at 30°C (for Hcp1-myc) or in LB media at 23°C (for Hcp2-myc). Expression (cells) and secretion (medium) of Hcp1-myc and Hcp2-myc were detected by immunoblot using anti-myc antibodies. Loading control (LC) is shown for total protein lysate. (**A**) POR1 or POR1Δ*opaR* strains containing either an empty vector or a vector for the expression of OpaR (pOpaR). (**B**) POR1 strains treated with the indicated concentrations of phenamil.

It has previously been suggested that the *V. parahaemolyticus* quorum sensing master regulator, OpaR, is a negative regulator of T6SS1 expression and a positive regulator of T6SS2 expression [30], [31]. The results shown in Figure 1 support this proposal, as under certain conditions Hcp1 was better expressed when the initial culture densities were lower, whereas Hcp2 was better expressed when the initial culture densities were higher. As the effect of OpaR on the secretion through the *V. parahaemolyticus* T6SSs was not previously tested, we set to determine whether OpaR also regulates T6SS1 and T6SS2 secretion. To this aim, we monitored the expression and secretion of Hcp1 and Hcp2 in the POR1 strain and in a POR1Δ*opaR* strain, containing either an empty expression vector or a vector encoding OpaR. As expected, both expression and secretion of Hcp1 were up-regulated in the POR1Δ*opaR* and down-regulated when *opaR* was overexpressed from a plasmid (Figure 2A, lanes 2,6 and 3,7, respectively). In contrast, deletion of *opaR* down-regulated the secretion of Hcp2, but only marginally affected Hcp2 expression levels (Figure 2A, lanes 2 and 6). In agreement with this result, over-expression of *opaR* up-regulated both the expression and the secretion levels of Hcp2 (Figure 2A, lanes 3,4,7,8). These results support the proposal that the quorum sensing regulator OpaR is a negative regulator of T6SS1, and a positive regulator of T6SS2.

The finding that quorum sensing affects activity of T6SS1 and T6SS2, prompted us to investigate how other major differences in the *V. parahaemolyticus* living environment and life styles, besides population densities, can affect T6SS activation. Recently, a global gene expression profiling of *V. parahaemolyticus* showed that surface sensing triggers a program of gene expression that promotes colonization and virulence [42]. Although no T6SS genes were transcriptionally regulated by surface sensing, we set out to investigate whether surface sensing can affect protein expression levels and secretion of Hcp1 and Hcp2. To test this, we monitored the expression and secretion of Hcp1 and Hcp2 in the POR1 strain grown with addition of increasing concentrations of the polar flagellar motor inhibitor, phenamil, to simulate surface conditions while bacteria are growing in liquid [42]. As shown in Figure 2B, both expression and secretion of Hcp1 were elevated by addition of phenamil to the growth media. However, secretion of Hcp2 was inhibited by addition of phenamil, while the total Hcp2 expression level was not affected (Figure 2B). These results indicate that T6SS1 expression and secretion are up-regulated upon surface sensing, while only T6SS2 secretion is down-regulated by surface sensing.

### *V. parahaemolyticus* T6SS1 Kills *Vibrio cholerae* Under Marine-like Conditions

**Figure 3:**
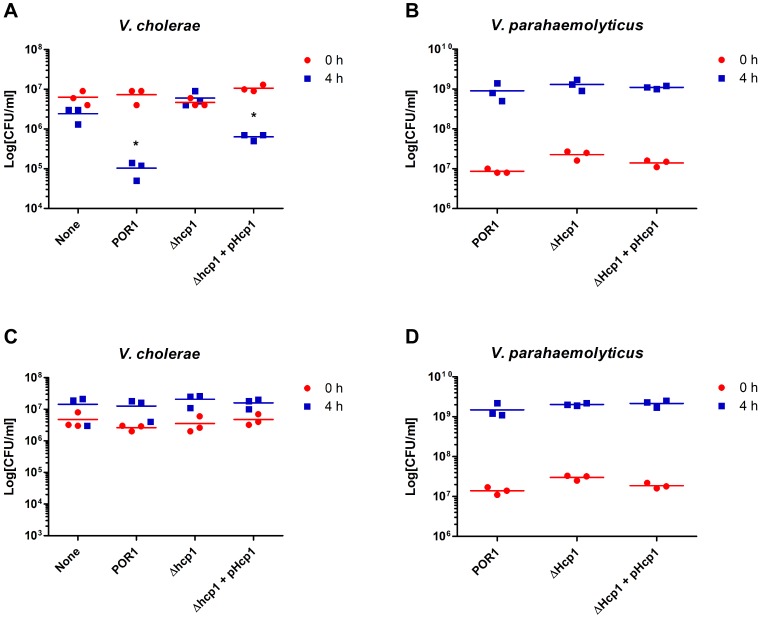
*V. parahaemolyticus* kills *V. cholerae* at 30°C using T6SS1. (**A–D**) Viability counts before (0 h) and after (4 h) co-culture of *V. cholerae* with MLB media (none) or *V. parahaemolyticus* POR1 or POR1Δ*hcp1* strains containing an empty vector or a vector encoding Hcp1 (pHcp1). Mixed cultures at an OD_600_ ratio of 1∶4 (*V. cholerae*:*V. parahaemolyticus*) were spotted on MLB plates at 30°C (**A–B**) or 37°C (**C–D**). An asterisk indicates statistically significant difference in mean values as compared to the co-culture with POR1Δ*hcp1* strain carrying an empty vector at the 4 hours time point, by an unpaired, two-tailed student’s t-test (p value <0.005).

We focused our studies on T6SS1 since it is induced under summer marine-like conditions in which *V. parahaemolyticus* thrive, and is associated predominantly with the clinical isolates of *V. parahaemolyticus* [8]. Since several T6SSs were reported to play a role in interbacterial competition [12], [43], we hypothesized that T6SS1 would have anti-bacterial activity. To test this, POR1, a POR1 derivative deleted for *hcp1* (POR1Δ*hcp1*), or POR1Δ*hcp1* in which *hcp1* was complemented on a plasmid (POR1Δ*hcp1*+pHcp1) were co-cultured with *Vibrio cholerae* in marine-like conditions where we had observed Hcp1 secretion (Figure 1). The POR1 strain was virulent toward *V. cholerae*, causing a ∼70 fold reduction in CFU per milliliter, relative to the initial CFU per milliliter of *V. cholerae* in the culture (Figure 3A). POR1 virulence was abrogated upon deletion of *hcp1*, and restored when *hcp1* was complemented from a plasmid, indicating that the *V. parahaemolyticus* virulence was mediated via T6SS1 (Figure 3A). Importantly, all the *V. parahaemolyticus* strains grew similarly on the MLB-agar plates, as well as in liquid MLB (Figure 3B and S3). To determine whether environmental conditions play a role in *V. parahaemolyticus* virulence, we repeated this experiment at 37°C, a temperature that does not induce T6SS1 (Figure 1). We observed that although *V. parahaemolyticus* growth was not affected (Figure 3D), it was no longer virulent toward *V. cholerae* under these conditions (Figure 3C), further supporting our hypothesis that induction of T6SS1 is important for interbacterial competition and required for killing *V. cholerae* in a co-culture.

### *V. parahaemolyticus* Selectively Kills Various Gram-negative Bacteria Using T6SS1

**Figure 4:**
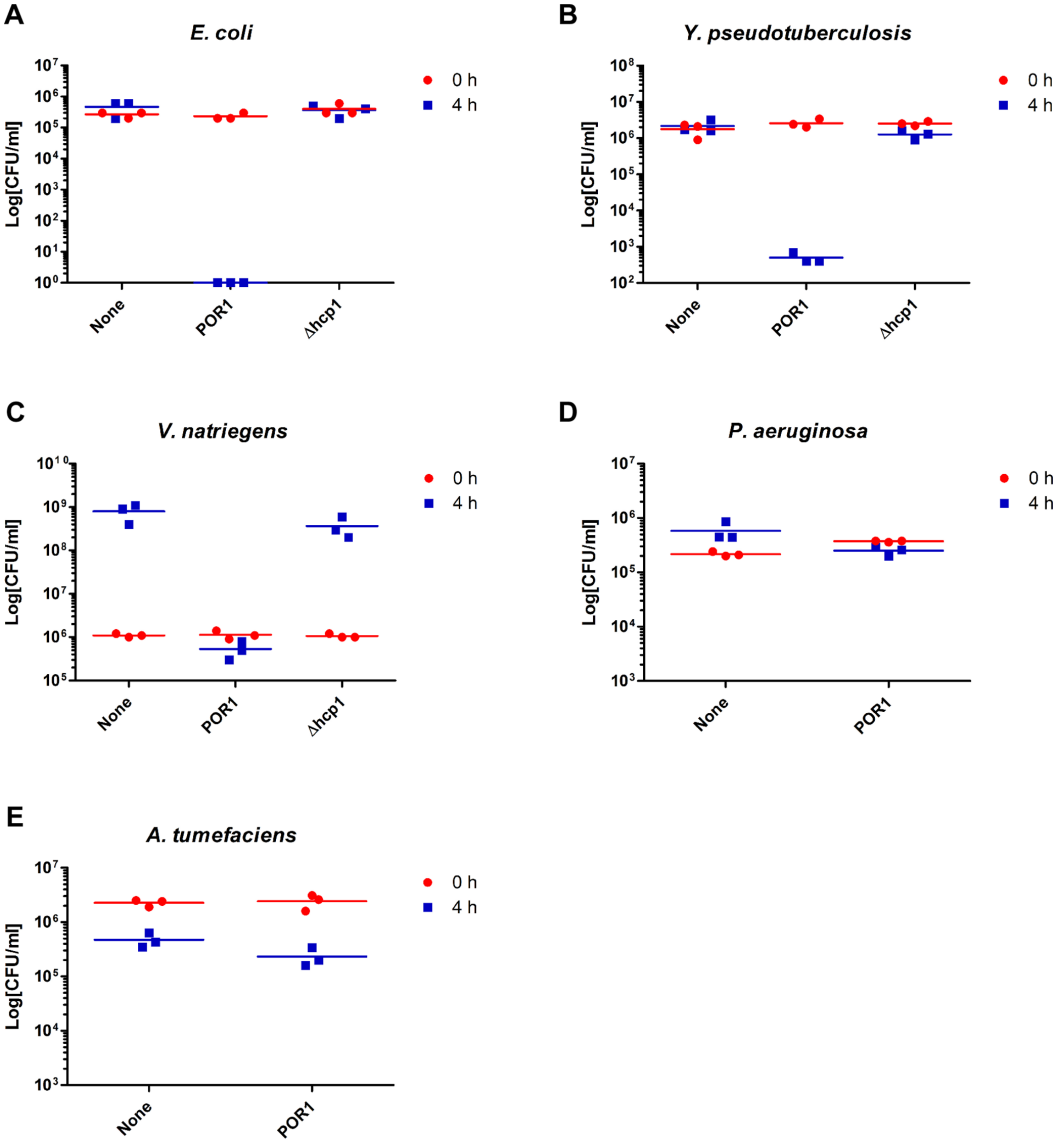
T6SS1 is toxic towards specific Gram-negative bacterial species. (**A–E**) Viability counts before (0 h) and after (4 h) co-culture of *E. coli* (**A**), *Y. pseudotuberculosis* (**B**), *V. natriegens* (**C**), *P. aeruginosa* (**D**) and *A. tumefaciens* (**E**) with MLB media (none) or *V. parahaemolyticus* POR1 or POR1Δ*hcp1* strains. Mixed cultures at an OD_600_ ratio of 1∶4 (prey bacteria :*V. parahaemolyticus*) were spotted on MLB plates at 30°C.

After showing that the *V. parahaemolyticus* T6SS1 functions in killing *V. cholerae*, we next determined the virulence of T6SS1 against other Gram-negative bacteria that *V. parahaemolyticus* might encounter in its different habitats or lifestyles. We screened five additional bacterial species and found that the POR1 strain is highly virulent towards *E. coli* (Figure 4A), *Yersinia pseudotuberculosis* (Figure 4B), and *Vibrio natriegens* when co-cultured under marine-like (T6SS1-inducing) conditions (Figure 4C). This virulence was dependent on T6SS1, as it was abolished in the POR1Δ*hcp1* strain. However, *Pseudomonas aeruginosa* and *Agrobacterium tumefaciens* were not susceptible to the anti-bacterial activity of T6SS1 (Figure 4D-E). All the bacterial species tested are of the γ-proteobacteria class, except for *A. tumefaciens* which is α-proteobacteria.

### VP1391 and VP1407 are Positive Regulators of T6SS1

**Figure 5:**
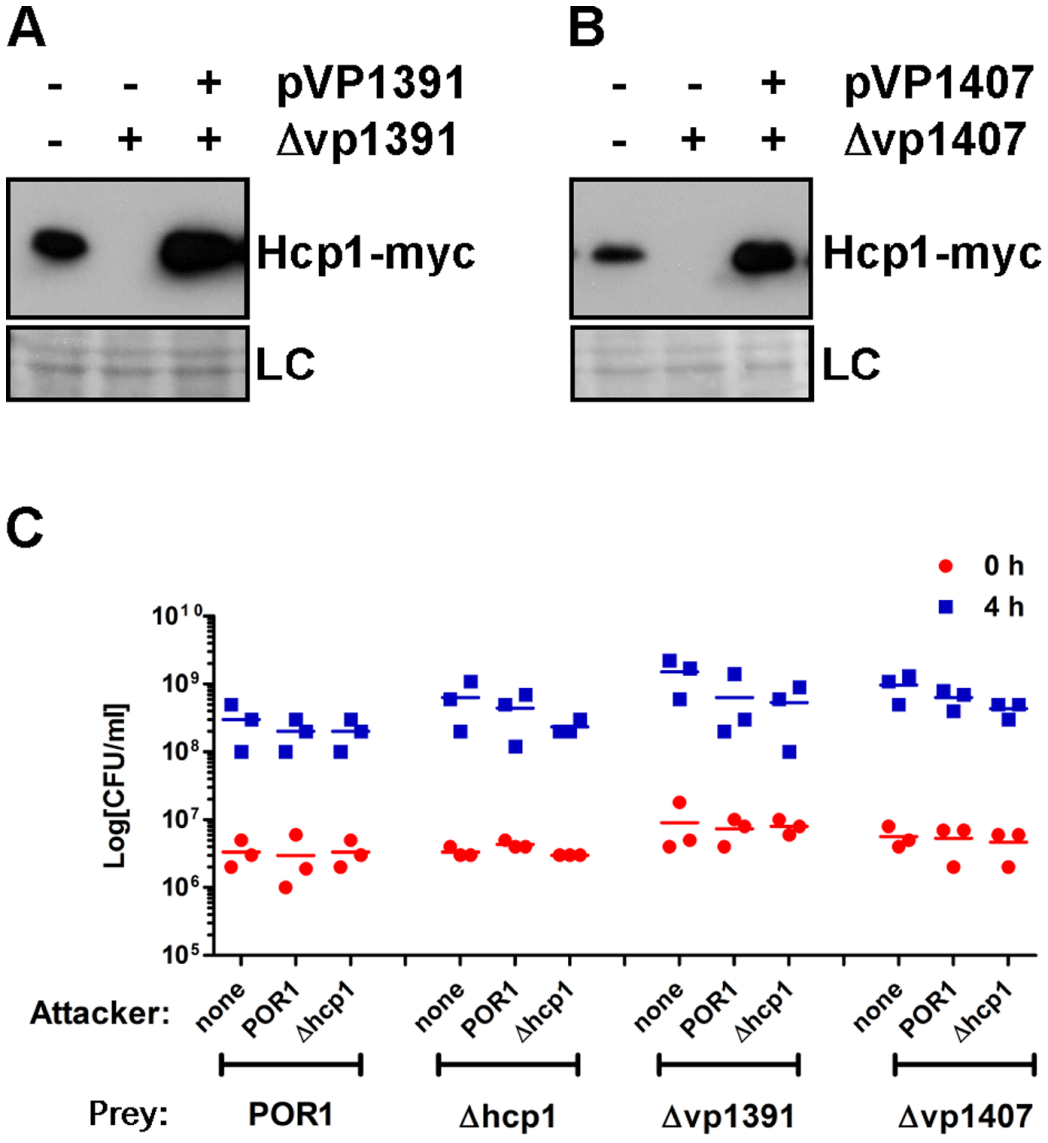
VP1391 and VP1407 are positive regulators of Hcp1 expression, but are not required for self-immunity. (**A–B**) Expression of endogenously C-terminal myc-tagged Hcp1 in *V. parahaemolyticus*. POR1 strains were grown in MLB media with 0.1% arabinose at 30°C and initial OD_600_ of 0.18. Strains contained either an empty expression vector or a vector encoding an arabinose inducible VP1391 (pVP1391) (**A**) or VP1407 (pVP1407) (**B**). Expression of Hcp1-myc was detected by immunoblot using anti-myc antibodies. Loading control (LC) is shown for total protein lysate. (**C**) Viability counts before (0 h) and after (4 h) co-culture of prey POR1 strains containing a pBAD vector, with MLB media (none) or *V. parahaemolyticus* POR1 or POR1Δ*hcp1* strains (attacker). Mixed cultures at an OD_600_ ratio of 1∶4 (prey:attacker) were spotted on MLB plates at 30°C.

The *V. parahaemolyticus* T6SS1 gene cluster encodes two putative transcription regulators, VP1391 and VP1407. VP1391 is predicted to be a member of the bacterial enhancer binding proteins (bEBPs) [44] and contains a sigma-54 interaction domain and a helix-turn-helix domain. Proteins belonging to this family are found in various T6SS gene clusters and were shown to regulate T6SS genes expression [45]. VP1407 is predicted to belong to the AsnC family of transcription regulators. To our knowledge, proteins from this family were not previously associated with T6SS regulation. To test if these two transcription regulators affect T6SS1, we monitored the expression of Hcp1 in POR1 derivative strains deleted for either *vp1391* or *vp1407*. Both VP1391 and VP1407 were required for expression of Hcp1 under marine-like conditions (Figure 5A–B), indicating that they are positive regulators of T6SS1.

Next, we asked whether *V. parahaemolyticus* is susceptible to its own T6SS1, and if not, are VP1391 or VP1407 required for immunity against self. The POR1 strain was not virulent toward itself and was not virulent toward a POR1Δ*hcp1* strain, suggesting that an active T6SS1 is not required for immunity against self (Figure 5C). Furthermore, POR1 was not toxic to POR1Δ*vp1391* and POR1Δ*vp1407* (Figure 5C), suggesting that while VP1391 and VP1407 regulate T6SS1, they are not required for immunity. Deletion of *vp1391* or *vp1407* did not affect *V. parahaemolyticus* growth (Figure S3).

### The Presence of T6SS1 Correlates with Anti-bacterial Virulence in Environmental Isolates

**Figure 6:**
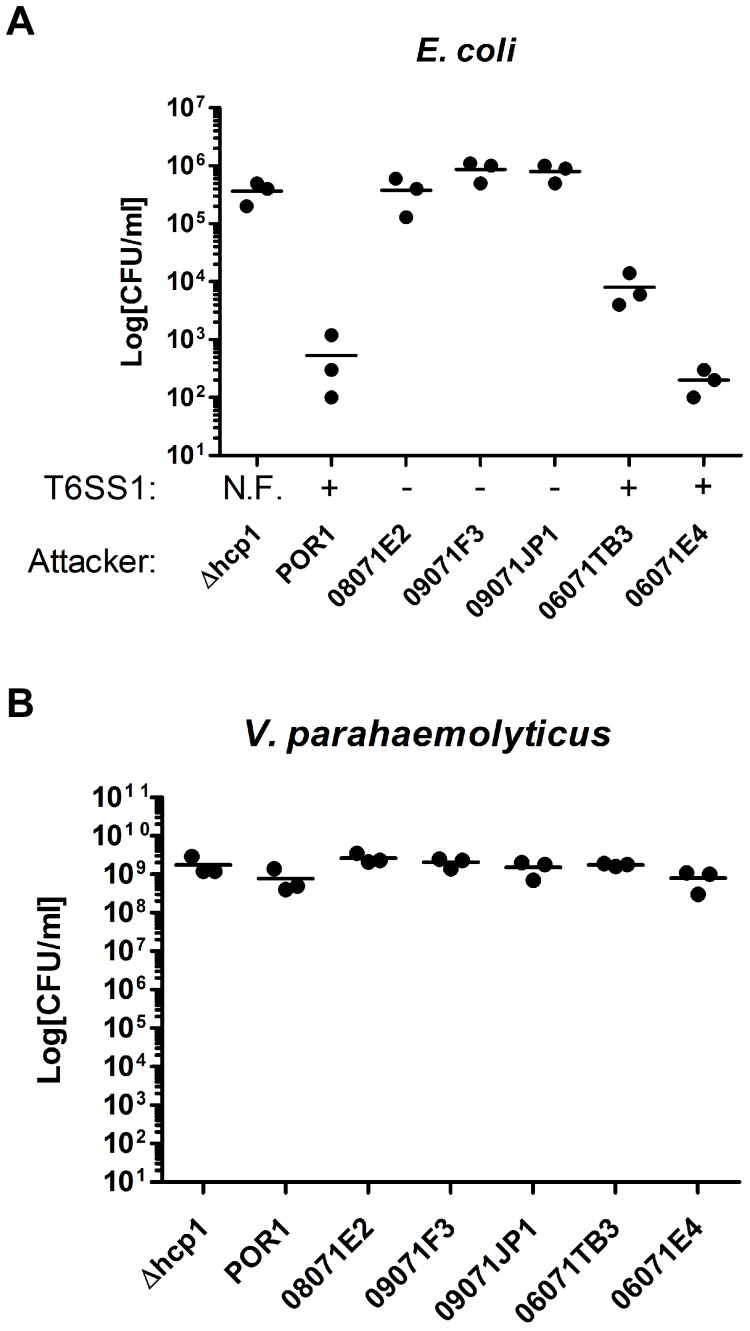
*V. parahaemolyticus* environmental isolates containing T6SS1 are virulent toward *E. coli*. (**A–B**) Viability counts after 4 hours of co-culture of *E. coli* (**A**) with the indicated *V. parahaemolyticus* strains (**B**). Mixed cultures at an OD_600_ ratio of 1∶4 (*E. coli*:*V. parahaemolyticus*) were spotted on MLB plates at 30°C. The presence (+) or absence (−) of the T6SS1 locus in *V. parahaemolyticus* strains is indicated. N.F. = non-functional.

Next, we examined environmental *V. parahaemolyticus* isolates from the North Inlet Estaury in Georgetown, South Carolina [46], for anti-bacterial virulence. Five *V. parahaemolyticus* isolates, 08071E2, 09071F3, 09071JP1, 06071TB3, and 06071E4 [46] were co-cultured with *E. coli* in marine-like conditions to test their virulence toward this bacterium. Three isolates (08071E2, 09071F3, and 09071JP1) were not virulent toward *E. coli* and behaved similarly to the non-virulent POR1Δ*hcp1* strain (Figure 6A). Two isolates (06071TB3 and 06071E4) were virulent toward *E. coli* and behaved similarly to the virulent POR1 strain (Figure 6A). Interestingly, based on PCR analysis of genomic DNA (Table S1), the two virulent isolates (06071TB3 and 06071E4) contained the T6SS1 genetic locus, whereas the three isolates that were not virulent toward *E. coli* (08071E2, 09071F3, and 09071JP1) did not contain T6SS1 (Figure 6A, and Table S1), providing a tentative correlation between the presence of T6SS1 and anti-bacterial virulence. Notably, the growth of all tested isolates was comparable to that of the POR1 strain (Figure 6B).

### POR1 is Virulent Toward Environmental *V. parahaemolyticus* Isolates in a T6SS1-dependent Manner

**Figure 7:**
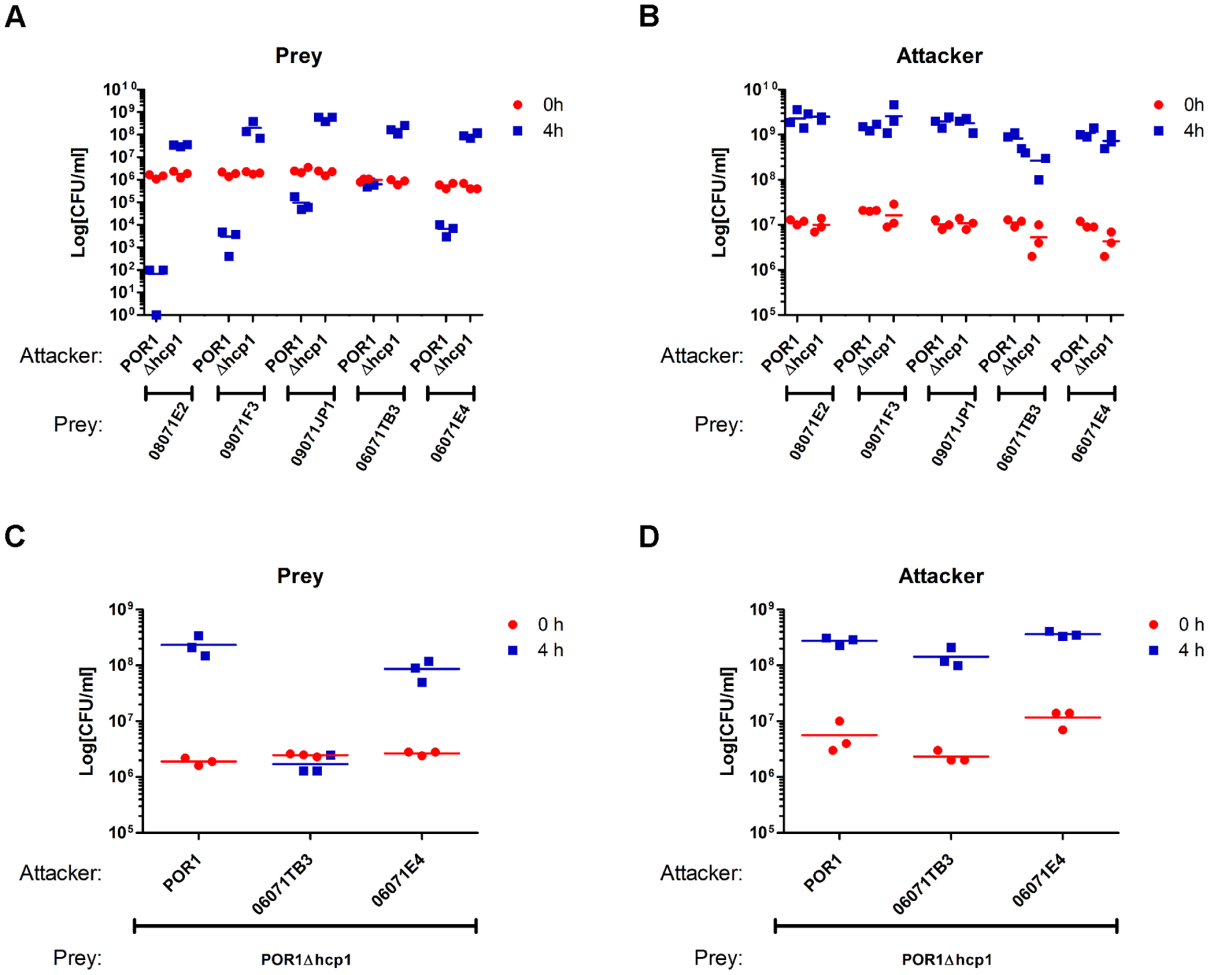
*V. parahaemolyticus* exhibit intra-species virulence. (**A–B**) Viability counts before (0 h) and after (4 h) co-culture of prey environmental *V. parahaemolyticus* isolates containing a pBAD33 vector (**A**), with attacker *V. parahaemolyticus* POR1 or POR1Δ*hcp1* strains containing an empty pBAD vector (**B**). (**C–D**) Viability counts before (0 h) and after (4 h) co-culture of prey POR1Δ*hcp1* strains containing an empty pBAD vector (C) with attacker T6SS1-positive environmental *V. parahaemolyticus* isolates containing a pBAD33 vector (**D**). Mixed cultures at an OD_600_ ratio of 1∶4 (prey:attacker) were spotted on MLB plates at 30°C.

As shown above, while the POR1 strain is virulent toward other bacterial species (Figure 3 and 4), it is unable to kill itself or its derivative strains with an inactive T6SS1 (Figure 5), presumably because it is immune against self-intoxication, as has been shown in other bacterial species containing T6SSs [12], [38]. To determine whether POR1 is capable of intra-species competition, we tested its virulence toward the environmental *V. parahaemolyticus* isolates described above. Remarkably, POR1 was virulent, to various degrees, toward all five isolates tested, regardless of whether they had the T6SS1 locus or were virulent themselves toward other bacteria (Figure 7A). The POR1 virulence was dependent on T6SS1, as virulence was abolished in the POR1Δ*hcp1* strain (Figure 7A). Notably, the growth of the *V. parahaemolyticus* attacker strains was comparable (Figure 7B). Moreover, when we tested the virulence of T6SS1-positive environmental *V. parahaemolyticus* isolates toward the POR1Δ*hcp1* strain, we observed a detrimental effect of the environmental strain 06071TB3 on POR1Δ*hcp1* growth (Figure 7C–D).

## Discussion

T6SSs are highly regulated systems used by Gram-negative bacteria for a variety of functions, including virulence and interbacterial competition [19], [43]. Most T6SSs studied to date are active under normal laboratory growth conditions, and the environmental conditions that regulate them are largely unidentified. Here, we investigated and characterized the environmental conditions and cues that regulate and activate the two T6SSs found in *V. parahaemolyticus.* We also identified a role for T6SS1 in interbacterial competition.

We found that the two *V. parahaemolyticus* T6SSs are differentially regulated by quorum sensing and by surface sensing, as T6SS1 is repressed by quorum sensing and induced by surface sensing, while these regulatory networks reciprocally regulate T6SS2. These results suggest that T6SS1 and T6SS2 act under considerably different sets of conditions, and therefore support the hypothesis that the two systems play different roles for *V. parahaemolyticus* varied lifestyles.

Interestingly, a previous study described a reciprocal effect of OpaR on the transcription of T6SS1 and T6SS2 genes [30]. OpaR was shown to be a positive transcriptional regulator of T6SS2 genes and a negative transcriptional regulator of T6SS1 genes [30]. We have examined the effects of OpaR on the protein level expression and secretion of Hcp1 and Hcp2 and found that in agreement with previous transcription results, OpaR negatively affects Hcp1 expression and secretion while positively affecting Hcp2 secretion. However, we did not observe an OpaR-mediated effect on Hcp2 protein expression levels, suggesting that the effect OpaR had on Hcp2 secretion was caused by its effect on expression of other T6SS2 components, likely the T6SS2 machinery. It should be noted that the effect of OpaR on Hcp1 expression was recently shown to be indirect [31].

The conditions that we have identified as regulating T6SS1 activity (*i.e.* positive regulation by surface sensing and negative regulation by quorum sensing) were previously shown to regulate expression of several other virulence determinants in a similar manner [30], [42]. For example, the cytotoxic T3SS1, as well as additional potential virulence factors such as collagenase, were shown to be up-regulated by surface sensing [42]. Moreover, the T3SS1, like T6SS1, is up-regulated in OpaR-silenced strains, and these deletion strains are more cytotoxic to mammalian cells than the strains expressing wild-type OpaR [30]. Therefore, it is possible that similar regulatory mechanisms control virulence against both eukaryotes and prokaryotes.

Our results also demonstrated that T6SS1 and T6SS2 are differentially regulated by temperature and salinity conditions, and while T6SS2 was only active under low salt conditions, T6SS1 was most active under high salt conditions at warm temperatures. The latter conditions mimic the conditions found during summer months in many coastal marine environments (according to the national oceanographic data center www.nodc.noaa.gov) in which high populations of *V. parahaemolyticus* and high infection rates were reported [2], [4], [46], [47], [48]. Interestingly, the average salinity in the marine and estaurine environments in which *V. parahaemolyticus* is found is 2–3% [43], [49], [50], while it is believed to be lower inside oysters and animal intestine [44]. Therefore, we hypothesize that T6SS1 functions when *V. parahaemolyticus* is in sea water, while T6SS2 functions when the bacterium is inside a marine animal.

Remarkably, neither of the T6SSs were active at 37°C. This supports a hypothesis that the *V. parahaemolyticus* T6SSs are not active at temperatures similar to those found in the digestive systems of land mammals, such as humans. Thus, the two T6SSs might not play a direct role in mammalian virulence. More likely, the regulatory mechanisms of the T6SSs have evolved to benefit this pathogen in the marine environment.

Nevertheless, the fact that T6SS1 is found predominantly in clinical isolates of *V. parahaemolyticus* [8] indicated that it may have a role in enhancing this bacterium’s environmental fitness. Indeed, we describe here a role for T6SS1 in interbacterial competition. *V. parahaemolyticus* was able to selectively kill several Gram-negative bacteria that were tested, including *E. coli*, *Y. pseudotuberculosis*, *V. cholerae*, *V. natriegens*, as well as other *V. parahaemolyticus*. This virulence was completely dependent on T6SS1. Notably, *V. parahaemolyticus* can encounter the various bacteria tested in its natural habitats. Like *V. parahaemolyticus,* strains of *E. coli*, *Y. pseudotuberculosis*, and *V. cholerae* can inhabit the animal gut [48], [51], [52]. *V. cholerae*, *V. natriegens*, and *P. aeruginosa* can be found in water environments together with *V. parahaemolyticus* [47], [53], [54]. *A. tumefaciens* are found in soil environments which can, on rare occasions, be occupied by *V. parahaemolyticus* [45], [55], [56]. Since T6SS1 was absolutely required for virulence against other bacteria, we suggest that T6SS2 does not play a role in interbacterial competition under the temperature, salinity, and surface conditions used in our assay.

*V. parahaemolyticus* virulence toward other bacteria was selective, as it was unable to kill *P. aeruginosa* and *A. tumefaciens*. This result could be explained in different ways: (1) *V. parahaemolyticus* targets a bacterial component that is not found or is not essential for some bacterial species; (2) some bacterial species can suppress T6SS1 activity; (3) some bacteria are immune to T6SS1 toxicity as was demonstrated previously with other systems [38]. Interestingly, *P. aeruginosa* PAO1 was not susceptible to anti-bacterial T6SS of other bacteria, such as *V. cholerae* [13], and *A. tumefaciens* was not susceptible to an anti-bacterial T6SS of *Burkholderia* [14], suggesting that these two bacterial species can protect themselves against a variety of T6SSs.

Notably, *V. cholerae*, *E. coli*, and *Y. pseudotuberculosis* do not proliferate on the MLB-agar plates used in our killing assays at 30°C. Since there is no change in bacterial counts from the beginning of the experiment to its end when these bacteria were grown in the absence of other bacteria, we can conclude that T6SS1 is in fact actively killing the target bacteria as opposed to just inhibiting proliferation. We observed the CFU per milliliter of the prey bacteria decline during co-culturing with POR1.

Interestingly, we showed that the POR1 strain was virulent toward several *V. parahaemolyticus* environmental isolates. As the POR1 strain was immune to self-intoxication, we conclude that the environmental isolates that were tested lack components that are required for immunity against a toxin(s) used by the T6SS1 found in POR1. Therefore, *V. parahaemolyticus* strains use different sets of T6SS effectors and immunity proteins. Furthermore, these results indicate that *V. parahaemolyticus* uses T6SS1 to compete against other bacterial species as well as against its own species. A similar observation was made recently for various *V. cholerae* strains [57].

Based on our results, we can suggest a possible environmental scenario in which *V. parahaemolyticus* uses T6SS1 to enhance its fitness. During the summer, when coastal waters are warm, there is an increase in marine bacterial populations, and therefore *V. parahaemolyticus* has to compete for a niche. Hence, single or few cells that land on a surface in the warm and salty ocean waters activate their T6SS1 to fight and kill foreign bacteria so that they can compete with them for resources. Moreover, under these conditions some strains can also use T3SS2 to fight off bacterivorous protists [58]. After the *V. parahaemolyticus* population established itself in a niche (and possibly formed a biofilm), there is no longer a need to activate the energy-consuming anti-bacterial T6SS1, and it is thus repressed by quorum sensing.

The identification of the anti-bacterial role of T6SS1 and the conditions under which it is active improves our understanding of mechanisms used by *V. parahaemolyticus* clinical strains to enhance their environmental fitness in mixed bacterial populations in marine environments. Future studies on T6SS1 and its targets can provide us with strategies to help in prevention of *V. parahaemolyticus* infections as well as infections by other *Vibrios*, such as *Vibrio alginolyticus*, which contains a similar T6SS.

## Materials and Methods

### Bacterial Strains and Media

The *Vibrio parahaemolyticus* RIMD 2210633 derivative strain POR1 (RIMD 2210633 Δ*tdhAS*) [6] and its derivatives, as well as *V. parahaemolyticus* environmental isolates [46], were routinely cultured in Marine Luria-Bertani (MLB) broth (Luria-Bertani broth containing 3% sodium chloride) or on Marine minimal media (MMM) agar [53] at 30°C. *E. coli* DH5α, *E*. *coli* Sm10 (λ pir), *Pseudomonas aeruginosa* PAO1, and *Vibrio cholerae* El Tor N16961, were routinely cultured in 2×YT broth at 37°C. *Vibrio natriegens* ATCC® 14048 and *Agrobacterium tumefaciens* GV2260 were routinely cultured in 2×YT broth at 30°C. *Yersinia pseudotuberculosis* YP37 [47] was routinely cultured in 2×YT broth at 23°C. The medium was supplemented with ampicillin (100 µg/ml), streptomycin (50 µg/ml), kanamycin (30 µg/ml or 120 µg/ml for *V. parahaemolyticus*), rifampicin (100 µg/ml), or chloramphenicol (25 µg/ml) where necessary.

### Construction of Deletion and Knock-in Strains

In-frame deletions of T6SS genes were performed by cloning the nucleotide sequences 1 kb upstream and 1 kb downstream of *opaR* (*vp2516*), *hcp1* (*vp1393*), *vp1391*, and *vp1407* into pDM4, a Cm^R^OriR6K suicide plasmid. In-frame C-terminal myc tag knock-in of *hcp1* and *hcp2* (*vpa1027*) were performed by cloning a C-terminal myc tagged version and the nucleotide sequences 1 kb downstream of *hcp1* or *hcp2* into pDM4. The resulting plasmids were conjugated into the POR1 strain from *E*. *coli* Sm10 (λ pir) and transconjugants were selected on media containing 25 µg/ml chloramphenicol. Bacteria were counter-selected by growing on media containing 15% sucrose. Deletions and insertions were confirmed by PCR.

### Plasmids Construction

For ectopic expression of OpaR and Hcp1, the genes coding sequences including 1 kb upstream were amplified and cloned into the pBAD/*Myc*-His vector (Invitrogen) in which the antibiotic resistance was changed from ampicillin to kanamycin. For arabinose-inducible ectopic expression of VP1391 and VP1407, the genes coding sequences were amplified and cloned into the pBAD2 vector (pBAD/*Myc*-His in which the NcoI restriction site had been removed). All genes were amplified with a stop codon to avoid the C-terminal tag found in the pBAD vectors.

### Identification of T6SS Genetic Loci

To determine whether environmental isolates of *V. parahaemolyticus* harbor T6SS1 and T6SS2 genetic loci, PCR was performed on genomic DNA to detect the presence of *vipA1* (*vp1402*) and *vipA2* (*vpa1035*), respectively. Primers used for amplification of the *vipA1* coding region were: 5′-CACGTGACGGCTCGGTGG-3′ and 5′-CTCTTCTTTCGCGTCTTGGTCG- 3′. Primers used for amplification of the *vipA2* coding region were: 5′-CGAGTATCCACTCGAAACTTTC-3′ and 5′-TTCTGCTCCCTCAGTACTTTCTG- 3′.

### Expression and Secretion Assays

Bacterial strains were grown overnight in MLB with appropriate antibiotics to maintain plasmids when necessary. 0.9 or 4.5 OD_600_ units were washed and re-suspended in 20 milliliter borosilicate glass tubes in 5 milliliters of LB or MLB (to produce cultures of OD_600_ = 0.18 and OD_600_ = 0.9, respectively) with appropriate antibiotics to maintain plasmids when necessary, and with 0.1% arabinose if induction of expression from a plasmid was required. Cultures were incubated with agitation (215 rpm) for 5 hours at 23°C, 30°C, or 37°C. OD_600_ values of the POR1 strain after 5 hours of growth under these conditions are shown in Table S2. For expression fractions (cells) 1.5 OD_600_ units were collected and cell pellets were re-suspended in 100 µl of 2X protein sample buffer (100 mM Tris pH = 6.8, 20% glycerol, 2% sodium dodecyl sulfate (SDS), 2% β-mercaptoethanol, 150 mM sodium hydroxide, bromophenol blue). Supernatants of 10 OD_600_ units were filtered and precipitated with deoxycholate and trichloroacetic acid [54]. Precipitated proteins were pelleted and washed with acetone, prior to re-suspension in 45 µl of 2X protein sample buffer. Expression and secretion samples were resolved on SDS-polyacrylamide gel electrophoresis (SDS-PAGE), transferred onto PVDF membranes, and immunoblotted with c-myc antibodies (Santa Cruz Biotechnology). Equal loading of total protein lysate was confirmed by analysis of representative bands using Ponceau S staining of the immunoblot membrane. Equal volumes of medium were used from cleared, centrifuged cultures for all TCA precipitations.

### Bacterial Killing Assay

Bacterial strains were grown over-night in MLB (*V. parahaemolyticus*) or 2×YT (*E. coli*, *Y. pseudotuberculosis*, *V. cholerae*, *V. natriegens*, *P. aeruginosa*, *A. tumefaciens*). Bacterial cultures were mixed at a 4∶1 ratio (attacker:prey) in triplicates after the cultures were normalized to OD_600_ of 0.5. 25 µl of the mixtures were spotted on MLB plates and incubated at 30°C or 37°C for 4 hours. CFU per milliliter of the attacker and prey spotted on MLB plates at t = 0 were determined by plating 10-fold serial dilutions on selective media plates. Bacterial spots were harvested from MLB plates after 4 hours and the CFU per milliliter of the surviving attacker and prey were determined by plating 10-fold serial dilutions on selective media plates. *V. parahaemolyticus* were selected on MMM plates, or MMM plates with kanamycin (200 µg/ml) for strains containing a pBAD plasmid, or MMM plates with chloramphenicol (25 µg/ml) for strains containing a pBAD33 chloramphenicol resistance plasmid; *V. natriegens* and *E. coli* containing the pBAD33 plasmid, *Y. pseudotuberculosis*, and *P. aeruginosa* were selected on LB with chloramphenicol (25 µg/ml) plates; *V. cholerae* were selected on LB with streptomycin (50 µg/ml) plates, *A. tumefaciens* were selected on LB with rifampicin (100 µg/ml) plates. An empty pBAD33 plasmid was used to provide chloramphenicol resistance to *E. coli*, *V. natriegens*, and *V. parahaemolyticus*. All bacterial killing assays were repeated at least twice with similar results, and results of a representative experiment are shown.

### Bacterial Growth Assay

Triplicates of over-night grown *V. parahaemolyticus* strains were normalized to OD_600_ = 0.1 in MLB, and growth was measured as OD_600_ at 30°C with agitation (215 rpm).

## Supporting Information

Figure S1 **Organization of the V.** ***parahaemolyticus*** **RIMD 2210633 T6SS gene clusters.** Genes discussed in this work are annotated. Genes that were used or manipulated in this study are in blue. (**A**) T6SS1 gene cluster. (**B**) T6SS2 gene cluster.
(TIF)

Figure S2 **Expression and secretion of Hcp1 are induced by salinity.** *V. parahaemolyticus* POR1 strain containing endogenously C-terminal myc-tagged Hcp1 was grown under the indicated media conditions at 30°C at initial OD_600_ of 0.18. Expression (cells) and secretion (medium) of Hcp1-myc were detected by immunoblot using anti-myc antibodies. Loading control (LC) is shown for total protein lysate.
(TIF)

Figure S3 **T6SS1 deletions do not affect** ***V. parahaemolyticus*** **growth.** Growth of POR1 and derivative strains in MLB at 30°C based on OD_600_ measurements. Data are mean ± SD, n = 3.
(TIF)

Table S1 Distribution of T6SSs in *V. parahaemolyticus* environmental isolates.
(DOCX)

Table S2 OD_600_ values for *V. parahaemolyticus* POR1 cultures after 5 hours incubation (mean ± SD, n = 4).
(DOCX)
