# Supplementary figures and images for: Vibrio parahaemolyticus Type VI Secretion System 1 Is Activated in Marine Conditions to Target Bacteria, and Is Differentially Regulated from System 2
Source: PLoS One. 2013 Apr 16;8(4):e61086. doi: 10.1371/journal.pone.0061086 (PMC3628861; doi:10.1371/journal.pone.0061086)

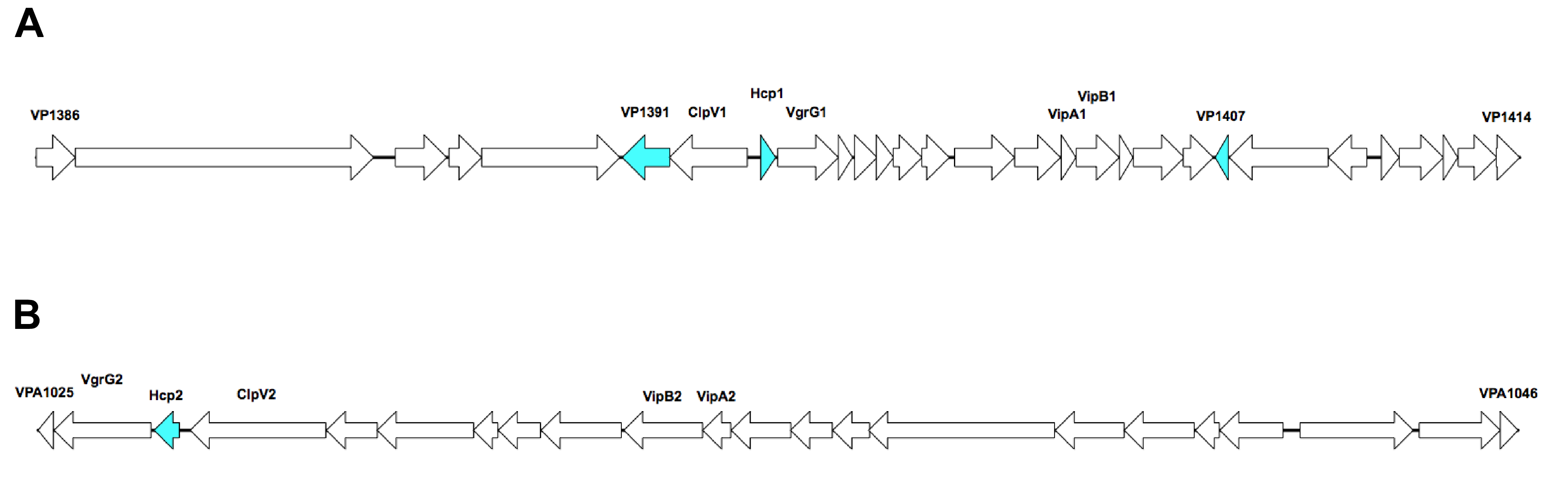

Supplement: Figure S1 — Organization of the V. parahaemolyticus RIMD 2210633 T6SS gene clusters. Genes discussed in this work are annotated. Genes that were used or manipulated in this study are in blue. (A) T6SS1 gene cluster. (B) T6SS2 gene cluster. (TIF) [file pone.0061086.s001.tif]

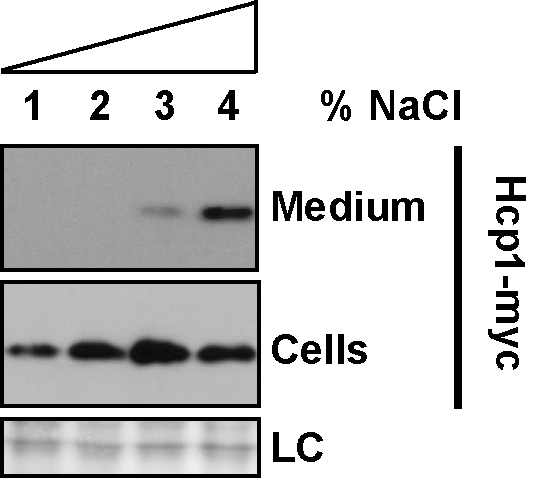

Supplement: Figure S2 — Expression and secretion of Hcp1 are induced by salinity. V. parahaemolyticus POR1 strain containing endogenously C-terminal myc-tagged Hcp1 was grown under the indicated media conditions at 30°C at initial OD600 of 0.18. Expression (cells) and secretion (medium) of Hcp1-myc were detected by immunoblot using anti-myc antibodies. Loading control (LC) is shown for total protein lysate. (TIF) [file pone.0061086.s002.tif]

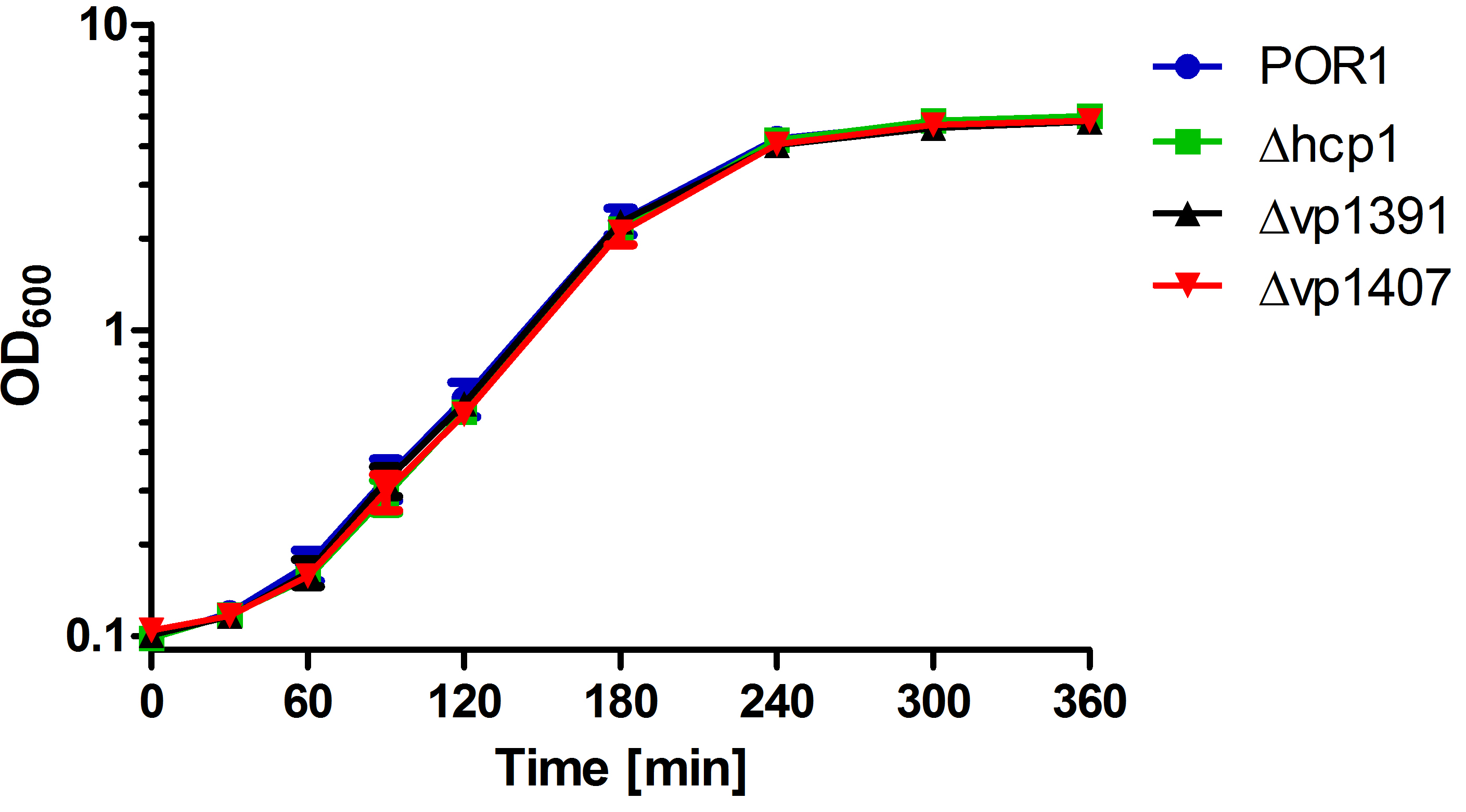

Supplement: Figure S3 — T6SS1 deletions do not affect V. parahaemolyticus growth. Growth of POR1 and derivative strains in MLB at 30°C based on OD600 measurements. Data are mean ± SD, n = 3. (TIF) [file pone.0061086.s003.tif]
