# Supplementary material for: Vibrio parahaemolyticus Type VI Secretion System 1 Is Activated in Marine Conditions to Target Bacteria, and Is Differentially Regulated from System 2
Source: PLoS One. 2013 Apr 16;8(4):e61086. doi: 10.1371/journal.pone.0061086 (PMC3628861; doi:10.1371/journal.pone.0061086)
Supplement: Table S1 — Distribution of T6SSs in V. parahaemolyticus environmental isolates. (DOCX) [file pone.0061086.s004.docx]

**Table S1. Distribution of T6SSs in *V. parahaemolyticus* environmental isolates.**

| Isolate | *vipA1* (T6SS1) | *vipA2* (T6SS2) |
| --- | --- | --- |
| 08071E2 | **-** | **+** |
| 09071F3 | **-** | **+** |
| 09071JP1 | **-** | **+** |
| 06071TB3 | **+** | **+** |
| 06071E4 | **+** | **+** |
