# Supplementary material for: Vibrio parahaemolyticus Type VI Secretion System 1 Is Activated in Marine Conditions to Target Bacteria, and Is Differentially Regulated from System 2
Source: PLoS One. 2013 Apr 16;8(4):e61086. doi: 10.1371/journal.pone.0061086 (PMC3628861; doi:10.1371/journal.pone.0061086)
Supplement: Table S2 — OD600 values for V. parahaemolyticus POR1 cultures after 5 hours incubation (mean ± SD, n = 4). (DOCX) [file pone.0061086.s005.docx]

**Table S2. OD_600_ values for *V. parahaemolyticus* POR1 cultures after 5 hours incubation (mean ± SD, n=4).**

| Media | Initial OD_600_ | 23°C | 30°C | 37°C |
| --- | --- | --- | --- | --- |
| MLB | **0.18** | 4.24 ± 0.17 | 4.64 ± 0.12 | 2.89 ± 0.25 |
| MLB | **0.90** | 4.90 ± 0.14 | 5.08 ± 0.12 | 3.38 ± 0.20 |
| LB | **0.18** | 3.66 ± 0.09 | 4.15 ± 0.14 | 3.08 ± 0.17 |
| LB | **0.90** | 4.12 ± 0.09 | 4.11 ± 0.02 | 3.30 ± 0.03 |
